# Impact of pre-surgical reduction methods on soft tissue healing and surgical timing in ankle fractures

**DOI:** 10.1007/s00590-025-04301-9

**Published:** 2025-05-19

**Authors:** Idan Strul, Oren Ben-Lulu, Ranin Simaan, Alexey Semenistyy, Ariel D. Levine

**Affiliations:** 1 Technion – Israel Institute of Technology, Haifa, Israel; 2 Bnai Zion Medical Center, Haifa, Israel

**Keywords:** Ankle fractures, Weber B/C, Quigley’s technique, Bayesian analysis

## Abstract

**Background:**

Early closed reduction and immobilization are essential in managing unstable ankle fractures to mitigate soft tissue swelling prior to surgery. This retrospective, single-center, preliminary study compares the effect of two reduction maintenance techniques, plaster U-splinting and Quigley’s skin traction suspension, on time to surgery.

**Methods:**

A retrospective observational review was conducted on 54 patients (aged 18–65) with unstable ankle fractures (Weber B/C). Surgical timing served as the dependent variable; reduction technique and patient variables were independent variables. Both Bayesian estimation and frequentist methods, including *t* tests and correlation analyses, were employed.

**Results:**

Patients treated with Quigley’s skin traction experienced shorter median times to surgery (5.3 days, SD = 2.8) compared to the U-splint group (10.7 days, SD = 3.9). This difference was statistically significant (*p* < 0.001). Bayesian analysis (posterior mean difference: 5.4 days, 95% CrI: 3.2–7.5; Bayes Factor = 12.6) supported these findings. However, patients in traction were hospitalized throughout, introducing inherent bias.

**Conclusions:**

Preliminary findings suggest that the inpatient use of Quigley’s technique, likely through continuous elevation and regular monitoring, was associated with shorter time to surgery, though causality cannot be established due to confounding. This advantage must be weighed against the costs of hospitalization and potential complications. More extensive, prospective studies with standardized follow-up and complication reporting are needed.

**Level of evidence:**

III.

## Introduction

Most orthopedic surgeons in emergency departments (EDs) treat ankle injuries daily [1–9] Such injuries can range from simple sprains requiring nothing more than Rest, Ice, Compression, and Elevation (RICE) for treatment to complex fractures requiring Open Reduction with Internal Fixation (ORIF) surgery. Lateral malleolus fracture, also known as Weber A (Lauge-Hansen type SA), is the most common, is generally regarded as stable, and is typically treated conservatively through closed reduction and splinting or a Jones dressing. Bi/tri malleolar fractures with syndesmotic and ligamental damage or Weber B and C fractures (Lauge-Hansen SER or PER) are regarded as unstable, and the definitive treatment of most of these fractures will be surgical via ORIF. An early closed reduction is critical because it reduces pain, swelling, and neurovascular compromise, allowing for better soft tissue healing. [1, 2, 5, 9] This is particularly relevant since early surgical timing has been shown to reduce the risk of surgical site complications such as wound dehiscence and infection [10–12]. Orthopedic surgeons implement a variety of techniques for closed reduction. One of these techniques is the Quigley technique, described in his 1959 article, where the patient’s lower limb is suspended in a stockinette, resulting in external rotation and abduction of the thigh, which causes the foot and ankle to fall into adduction, internal rotation, and supination, essentially reversing the mechanism of injury [9].

**Fig. 1:**
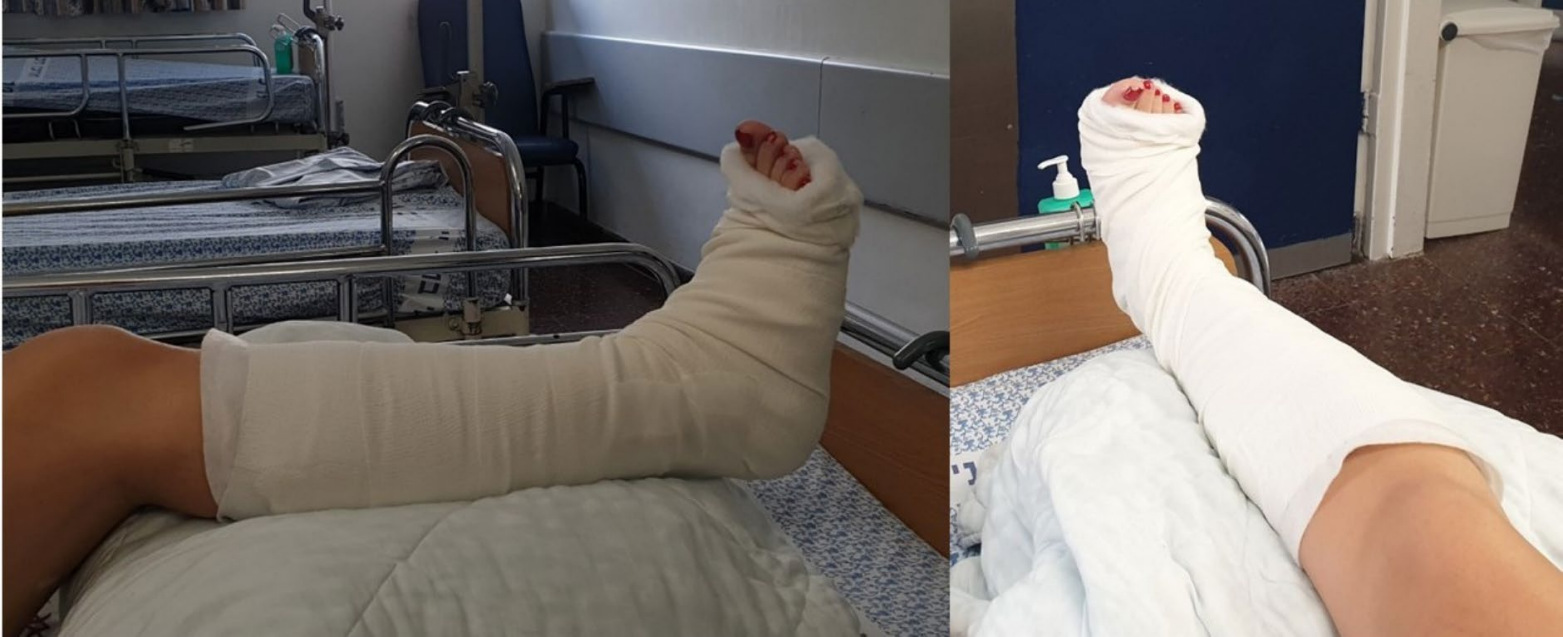
Two views demonstrating cast splinting as a treatment option while awaiting definitive surgery

Following a successful reduction, and depending on the fracture characteristics, several techniques, such as different casting methods and Jones dressing, etc., may be implemented to maintain the reduction. Casting using a U-splint or a full-leg cast is the most prevalent (see Fig. 1). However, recent literature has also emphasized the importance of fracture-specific management approaches, integrating patient comorbidities, fracture biomechanics, and post-operative mobilization strategies [13, 14].

**Fig. 2:**
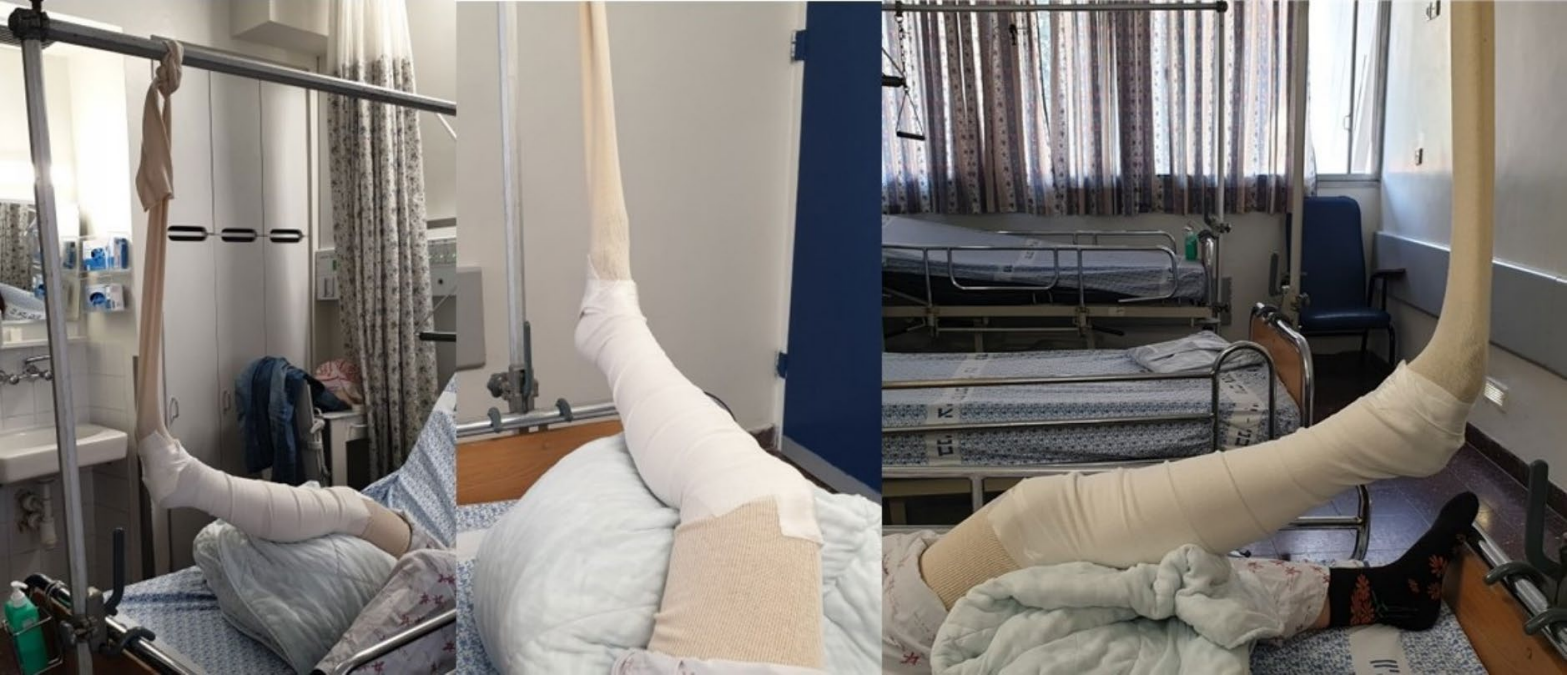
Three views demonstrate the stockinette suspension implementation in our department awaiting definitive surgery. Notice the position of the foot and ankle in the middle view, which reflects the adduction, internal rotation, and supination described by Quigley

An alternative is to keep the lower limb in a stockinette suspension. This technique is advantageous because maintaining reduction while the patient’s leg is constantly elevated allows for faster edema absorption, with fewer blisters, and may lead to earlier surgical treatment (see Fig. 2). However, this is not a stable splinting technique and requires hospitalization of the patient until definitive surgical treatment can be performed. Casting can rigidly hold the foot and ankle in position after a successful reduction, allowing for patient mobilization (either with crutches or a wheelchair) and discharge for outpatient follow-up until definitive surgical treatment. However, casting may be a double-edged sword since the patient's leg is not always elevated, leading to later surgical treatment due to slower soft tissue healing and a higher rate of edema and blisters [1, 5, 9].

Our primary objective in this research is to investigate which post-reduction pre-surgical treatment method yields faster soft tissue healing and fewer blisters, thereby enabling earlier definitive surgical treatment.

## Materials and methods

We conducted a single-center, observational, retrospective study using medical records. The files collected include all patients aged 18–65 who suffered from Weber B or C fractures and were operated on at the orthopedic surgery department at the authors’ medical center between 2019 and 2021. The collected data included fracture type, post-reduction treatment method, and time to definitive surgical treatment. We also collected Patients’ demographics, background diseases, functional status before the fracture, and inflammation indices on arrival to the E.D. All surgical procedures were performed by board-certified orthopedic trauma surgeons at the authors medical center.

Since this is a retrospective study, there is no concern about dropouts. However, there is potential selection bias since only patients who underwent surgery are included in this research, and only those who received the procedure at the authors’ medical center’s Orthopedic Surgery Department, not in the community. The study included fifty-four patients. The low number of patients may limit the statistical analysis’s power.

The Helsinki committee of the authors’ medical center approved this study.

### Research variables

The dependent variable in this study is the time between the fracture and the definitive operation. The independent variables in this study are:

- Pre-surgical reduction technique
- Background diseases
- Functional status before the fracture
- Inflammation indices around the day of surgery

### Minimal sample size

We expect a 15% difference between the Quigley technique and casting time for surgery. We implement a two-sided hypothesis. α is expected to be 5%, and power = 80% (1 − *β*). The minimal sample size in both groups is 50. However, real-world constraints and retrospective inclusion criteria limited enrollment to 35 and 19 patients in the U-splint and Quigley groups, respectively. This shortfall is further discussed in the discussion section.

### Statistical analysis

We employed a hybrid approach combining both frequentist and Bayesian methods. Initially, descriptive statistics were used to summarize group characteristics. Inferential analysis was conducted using t tests for group comparisons and ANCOVA to adjust for age, fracture type, and CRP. We also implemented Bayesian estimation to support findings in the context of a small sample. Posterior mean differences and credible intervals (CrI) were calculated using Bayesian linear modeling.

## Results

### Patient demographics

We divided the patients into two different groups according to the post-reduction technique implemented before definitive surgery: (1) the U-splint casting technique and (2) stockinette suspension (Quigley technique). None of the patients in the Quigley group received casting after reduction; the suspension method was used exclusively throughout their preoperative period. The median age of the U-splint group was 40.1 years with a standard deviation (SD) of 14.0, while the median age for the Quigley technique group was 44.9 years with a standard deviation of 14.7 (see Table 1). Age did not differ between treatment groups, nor were there any significant differences between groups in terms of gender.

**Table 1:** Frequencies of background diseases

	U-splint (*n* = 35)	Quigley (*n* = 19)	*p* value
Median age [years]	40.1 ± 14.0	44.9 ± 14.7	*p* > 0.05
Male (%)	54.3	36.8	*p* > 0.05
Hypertension (%)	22.857	15.789	*p* > 0.05
Diabetes mellitus (%)	11.428	15.789	*p* > 0.05
Osteoporosis (%)	2.857	10.526	*p *> 0.05
Hyperlipidemia (%)	11.428	15.789	*p* > 0.05
Hypothyroidism (%)	5.714	10.526	*p* > 0.05
Obesity (%)	8.571	10.526	*p* > 0.05
Alcohol use (%)	5.714	0	*p* > 0.05
Tabaco use (%)	5.714	21.052	*p* > 0.05
None of the above (%)	54.285	42.105	*p* > 0.05

While individual *p* values were not displayed for each comorbidity, all were assessed using Fisher’s exact test and found to be statistically non-significant (*p* > 0.05). Regarding the distribution of background diseases between the two groups

A notable discrepancy was observed in tobacco use: 21.1% in the Quigley group vs. 5.7% in the U-splint group. While this difference did not reach statistical significance (*p* > 0.05), it could potentially confound results. However, tobacco is a known risk factor for impaired wound healing, yet the Quigley group demonstrates a shorter time to surgery, as shown below. This warrants closer analysis in future prospective trials.

As for background diseases that could influence survival, we examined several common conditions that may affect the time between reduction and definitive surgery: hypertension, diabetes mellitus without mention of complications, osteoporosis, hyperlipidemia, hypothyroidism, obesity, alcohol use, and tobacco use. Nineteen patients in the U-splint group had none of these conditions, compared to only eight patients in the Quigley technique group. We found no statistically significant differences in the distribution of background diseases (see Table 1).

The median C-reactive protein (CRP) level in the U-splint group was 9.4, with a standard deviation of 10.7. In contrast, the median CRP level for the Quigley technique group was 16.0, with a standard deviation of 21.8 (see Table 2). CRP did not differ between treatment groups. CRP was recorded to evaluate systemic inflammatory response, which may correlate with soft tissue readiness for surgery, although no diagnostic thresholds were applied in this cohort. Regarding the Weber fracture type, 84.8% of patients in the U-splint group suffered from Weber B, while 15.2% suffered from Weber C fracture. In the Quigley technique group, 63.2% of patients suffered from a Weber B fracture, while 36.8% suffered from a Weber C fracture (see Table 2). There were no significant differences between groups on Weber category frequencies.

**Table 2:** First row CRP characteristics of the study population

	U-splint (*n* = 35)	Quigley (*n* = 19)	*p* value
CRP (mg/L)	9.4 ± 10.7	16.0 ± 21.8	*p* > 0.05
Weber B %	84.8	15.2	*p* > 0.05
Weber C %	63.2	36.8	*p* > 0.05

Second and third-row frequencies of Weber fracture type. No statistically significant difference in CRP or Weber fracture type was observed between the two groups *p* > 0.05

**Fig. 3:**
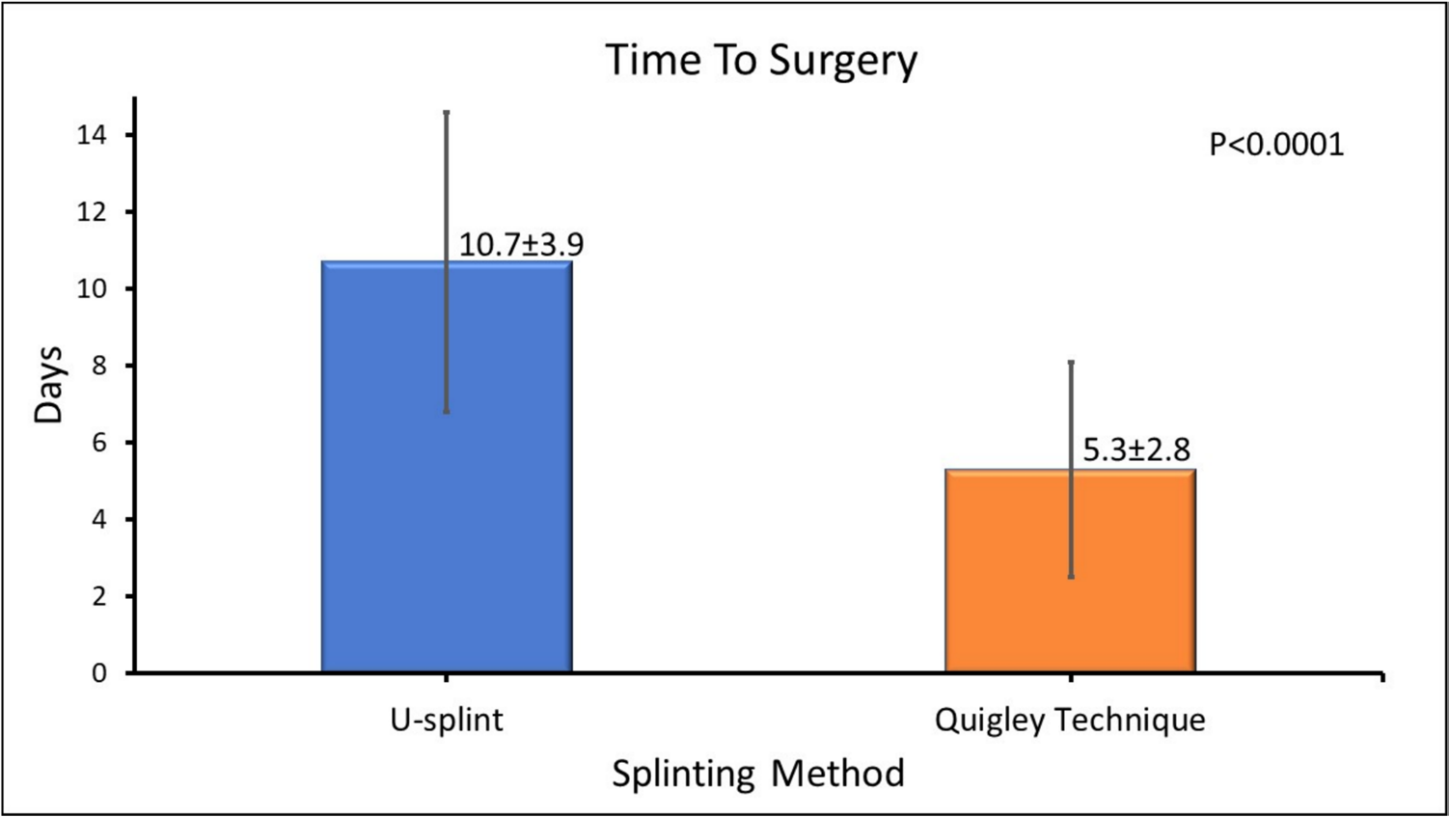
Bar graph illustrating the median number of days to surgery for the different splinting techniques. Blue—U-splint = 10.7 days with an SD of 3.9, orange—Quigley technique = 5.3 days with an SD of 2.8. The difference is statistically significant, *p* < 0.0001 (colour figure online)

The median days to surgery for the U-splint group was 10.7 days (IQR 8.4–13.2), while for the Quigley group, it was 5.3 days (IQR 4.0–6.8) (see Fig. 3). Measures of variability are now presented as interquartile ranges (IQR), which is appropriate for nonparametric data.

## Discussion

One of the most common fractures that orthopedic surgeons treat is ankle fractures. This study compared two post-reduction splinting techniques in patients with Weber B or C ankle fractures prior to definitive surgery. To our knowledge, no previous study has compared these two post-reduction pre-surgical techniques to determine which is more beneficial for patients with Weber B or C fractures, regarding soft tissue healing, timing of surgery, pain control, and rehabilitation time following surgery.

In our study, we found that age, gender, Weber fracture type, and CRP levels did not differ significantly between the two study groups in terms of the time between fracture reduction and definitive surgery.

However, we found that the Quigley technique is significantly superior to the U-splint plaster technique in terms of the time between reduction and definitive surgery. The U-splint plaster group had substantially more days to surgery than the Quigley group (t(52) = 5.3, *p* < 0.001). Furthermore, the treatment differences also remained significant after statistically controlling for the effects of age, CRP, and Weber fracture type (F(1,46) = 23.0, *p* < 0.001). It should be noted that all patients included in the study had successful reductions confirmed by post-reduction imaging. Therefore, no difference in initial reduction success was recorded between groups. Future studies may focus on maintenance integrity over time.

The strengths of this study include neutralizing many diseases that are known to affect the healing of soft tissues. Thus, the reduction technique would be the only explanation for the difference in the time between the reduction and definitive surgery. Moreover, the variability of the Israeli population is reflected in the two study groups, thus increasing the likelihood that the outcome is suitable for various patients.

As for limitations, the primary limitation of this study is its small sample size, which makes it challenging to determine whether the outcome is a factual finding and may lead to a Type II error. Another possible limitation of the study is the potential for selection bias in the Quigley group. The Quigley technique requires hospitalization, and the patients are examined daily during rounds, allowing for early identification of surgery-ready patients. However, most splinted patients returned to the outpatient clinic for follow-up within four to five days, with the intention of undergoing same-day surgery if soft tissue healing was sufficient. Moreover, patients in the U-splint plaster group, who can ambulate with crutches or wheelchairs, only elevate their braced leg for a few hours of the day. Hence, soft tissue healing and definitive surgery are delayed. This reduction in the time to surgery holds profound implications, as it means earlier mobilization, less muscle wasting, reduced anti-coagulation, and lower consumption of pain medication. Moreover, ankle fractures have significant economic implications. The direct costs within the health care system encompass expenses covered by insurers, including physician fees, diagnostic tests, and hospital stays. Meanwhile, patients face additional out-of-pocket costs for services not covered, such as transportation and personal expenses, in addition to productivity losses stemming from missed work or school and reduced participation in sports. These fractures are not merely fractures; they are a significant financial hurdle with far-reaching consequences. Hence, allowing for earlier surgical intervention and a return to activity is crucial.

If the Quigley technique halves the time to surgery, why is it not implemented broadly? As we stated, this approach requires hospitalization, which is expensive, and most health insurance providers may not justify the cost–benefit ratio. However, this raises the need to offer patients in-home limb suspension methods or explore new, reliable compression fixation methods, allowing them to enjoy the benefits of earlier surgery without the burden and cost of hospitalization. However, no follow-up was conducted to monitor complications such as wound dehiscence, infections, venous thromboembolism (VTE), pneumonia, or pressure ulcers. While the authors department has a standard anti-coagulation regime, patient adherence to it was not documented. These omissions limit conclusions regarding safety and should be addressed in future studies.

In conclusion, our study demonstrates a statistically significant difference in time between the reduction of Weber B or C ankle fractures and the timing of definitive surgery, comparing the Quigley reduction technique with the plaster U-splint. A more extensive sample size study should be conducted to determine if this outcome remains valid on larger scales. If replicated, our current splinting and outpatient follow-up methods should be revised to enable patients to benefit from earlier surgical intervention.

## Data Availability

Data will be made available upon request.
